# Consensus-based antimicrobial resistance and stewardship competencies for UK undergraduate medical students

**DOI:** 10.1093/jacamr/dlaa096

**Published:** 2020-12-04

**Authors:** David McMaster, Molly Courtenay, Catherine Santucci, Angharad P Davies, Andrew Kirby, Owen Seddon, David A Price, Gavin Barlow, Felicia H Lim, Bethany S Davies, Matthew K O’Shea, Paul Collini, Marina Basarab, Afshan Ahmad, Mahableshwar Albur, Carolyn Hemsley, Nicholas M Brown, Ciaran O’Gorman, Riina Rautemaa-Richardson, Geraint R Davies, Christopher N Penfold, Sanjay Patel, Keep Antibiotics Working (KAW) group, Afshan Ahmad, Andrew Kirby, Angharad P Davies, Bethany S Davies, Brian D Robertson, Carolyn Hemsley, Catherine Santucci, Christopher N Penfold, Ciaran O’Gorman, David A Price, David McMaster, Deborah Mitcheson, Elizabeth Hart, Felicia H Lim, Gavin Barlow, Geraint R Davies, Mahableshwar Albur, Marina Basarab, Matthew K O’Shea, Molly Courtenay, Nicholas M Brown, Nicola Jones, Owen Seddon, Patricia McGettigan, Paul Collini, Peter Munthali, Riina Rautemaa-Richardson, Sanjay Patel, Sophie Roberts, Tim Sloan, Timothy Paget

**Affiliations:** 1 University of Nottingham School of Medicine, Nottingham, UK; 2 Cardiff University School of Healthcare Sciences, Cardiff, UK; 3 Barts and the London School of Medicine and Dentistry, Victoria, Malta; 4 Swansea University Medical School, Swansea, UK; 5 Leeds Institute of Medical Research, The University of Leeds, Leeds, UK; 6 Medical Microbiology and Infectious Diseases, Public Health Wales, Cardiff, UK; 7 Department of Infection and Tropical Medicine, Newcastle upon Tyne Hospitals NHS Foundation Trust, Newcastle upon Tyne, UK; 8 Experimental Medicine and Biomedicine, Hull York Medical School, University of York, York, UK; 9 Department of Clinical Microbiology, University Hospitals of Leicester NHS Trust, Leicester, UK; 10 Department of Global Health and Infection, Brighton and Sussex Medical School, Brighton, UK; 11 Institute of Immunology and Immunotherapy, College of Medical and Dental Sciences, University of Birmingham, Birmingham, UK; 12 Department of Infection, Immunity and Cardiovascular Disease, University of Sheffield Medical School, Sheffield, UK; 13 Department of Infection, St George’s University Hospitals NHS Foundation Trust, London, UK; 14 Aston Medical School, Aston University, Birmingham, UK; 15 Severn Infection Sciences, North Bristol NHS Trust, Bristol, UK; 16 Department of Infectious Diseases, Guy’s and St Thomas’ NHS Foundation Trust, London, UK; 17 Clinical Microbiology and Public Health Laboratory, Cambridge University Hospitals NHS Foundation Trust, Cambridge, UK; 18 Centre for Medical Education, Queen’s University Belfast, Belfast, UK; 19 Department of Infectious Diseases, Manchester University Hospitals NHS Foundation Trust, Manchester, UK; 20 Institute of Infection and Global Health, University of Liverpool, Liverpool, UK; 21 School of Life Sciences, University of Nottingham, Nottingham, UK; 22 Department of Paediatric Infectious Diseases and Immunology, University Hospital Southampton NHS Foundation Trust, Southampton, UK

## Abstract

**Background:**

In the UK there is limited coverage of antimicrobial stewardship across postgraduate curricula and evidence that final year medical students have insufficient and inconsistent antimicrobial stewardship teaching. A national undergraduate curriculum for antimicrobial resistance and stewardship is required to standardize an adequate level of understanding for all future doctors.

**Objectives:**

To provide a UK national consensus on competencies for antimicrobial resistance and stewardship for undergraduate medical education.

**Methods:**

Using the modified Delphi method over two online survey rounds, an expert panel comprising leads for infection teaching from 25 UK medical schools reviewed competency descriptors for antimicrobial resistance and stewardship education.

**Results:**

There was a response rate of 100% with all 28 experts who agreed to take part completing both survey rounds. Following the first-round survey, of the initial 55 descriptors, 43 reached consensus (78%). The second-round survey included the 12 descriptors from the first round in which agreement had not been reached, four amended descriptors and 12 new descriptors following qualitative feedback from the panel members. Following the second-round survey, a total of 58 consensus-based competency descriptors within six overarching domains were identified.

**Conclusions:**

The consensus-based competency descriptors defined here can be used to inform standards, design curricula, develop assessment tools and direct UK undergraduate medical education.

## Introduction

Antimicrobial resistance (AMR) is one of the greatest threats to the future of healthcare.^1^ AMR occurs when microorganisms are exposed to antimicrobial drugs, with the misuse and overuse of antimicrobials accelerating the development of resistance.^1^ Infection with resistant microorganisms can have severe consequences, increasing mortality, prolonging hospital stays, adding a significant economic burden and threatening to undermine the global health improvements made over recent decades.^2^^,^^3^

Antimicrobial stewardship (AMS) is a coherent set of actions which promote responsible use of antimicrobials and is recognized as essential for limiting AMR.^4^ The WHO Global Action Plan on AMR emphasizes the importance of including AMS and antimicrobial prescribing in the training of health workers, calling for increased awareness and training by making AMR a core component of professional education.^2^ A variety of factors can result in injudicious use of antimicrobials by health workers, including fundamental lack of knowledge and awareness of AMR. In the UK there is inadequate coverage of AMS across the majority of postgraduate clinical training curricula, including specialities responsible for the largest volumes of antimicrobial usage (e.g. primary care) and hospital specialities which have high rates of broad spectrum antimicrobial use and healthcare-associated infections.^5^ The UK Foundation Programme curriculum, General Medical Council outcomes for graduates and the recently produced Royal College of Pathologists undergraduate curriculum are helpful guidance, but are unable to address AMR/S in sufficient detail to guide educators or standardize competencies.^6–8^

Education on AMR/S must be improved for all prescribers, including pharmacists, midwives, nurses, allied health professionals and doctors.^9–11^ Medical school prepares doctors for clinical practice, however there is evidence that final-year medical students have insufficient prescribing competencies and lack confidence in correctly prescribing antibiotics, despite these being among the most common medications junior doctors prescribe.^12–18^ Competencies represent a combination of knowledge, attitudes and skills and are designed to define the minimum standards that should be reached to practise responsibly and safely.^9^ Whilst AMR/S prescribing competency frameworks exist,^9–11^^,^^19^ there are no consensus-based AMR/S competencies for UK medical student education.

As part of the Keep Antibiotics Working (KAW) undergraduate programme, a joint BSAC, Health Education England and Medical Schools Council initiative was developed with the aim of providing a national consensus of competencies on AMR/S for UK undergraduate medical students.

## Methods

### Ethics

The research was conducted in accordance with the Declaration of Helsinki and national and institutional standards. Ethical approval was granted by the School of Healthcare Sciences Research Governance and Ethics Committee, Cardiff University (reference number 427).

### Delphi method

Using the modified Delphi method,^20^ the opinions of experts were gathered over two rounds of data collection. In collaboration with the Medical Schools Council, 28 experts (12 female; 16 male) were identified from 25 UK medical schools in England, Wales and Northern Ireland (Appendix S1, available as Supplementary data at *JAC-AMR* Online). After discussion with the Scottish Antimicrobial Prescribing Group, the decision was made to not include Scottish medical schools to avoid multiple simultaneous quality improvement initiatives. An expert in this study was defined as an individual from each institution with expertise in antimicrobial prescribing and medicines management, AMR/S and leading infection education for undergraduate medical students. Experts came from backgrounds in infectious diseases or microbiology and all were involved in leading undergraduate teaching. All experts were sent a participant information sheet and given the opportunity to discuss any queries with a researcher. All 28 experts agreed to participate.

A comprehensive list of core competencies was generated from available frameworks,^9^^,^^11^^,^^19^ allowing use of the modified Delphi method due to the availability of pre-existing information.^20^ These competencies were split into six domains, with overarching competency statements and a list of 55 descriptors designed to represent the knowledge, skills, attitudes and values that are required for undergraduate medical students (Table 1). As the competency frameworks used to generate the first-round survey had not been created for UK medical students it was important to include all descriptors for review in the first round.

**Table 1.** Round one responses

Competency descriptors	Median	IQR
Domain 1: Infection prevention and control		
1.1 Describing the nature and classification of pathogenic microorganisms	5	1
1.2 Describing how microorganisms cause infections in humans: the importance of understanding the differences between colonization (e.g. of venous leg ulceration) and infection	6	1
1.3 Explaining what an antimicrobial resistant organism is	6	1
1.4 Explaining the ‘Chain of Infection’	5	2^b^
1.5 How microorganisms are transmitted in both community and hospital settings	5	1
1.6 Defining the components required for infection transmission (i.e. presence of an organism, route of transmission of the organism from one person to another, a host who is susceptible to infection)	5	1
1.7 Describing the routes of transmission of infectious organisms (i.e. contact, droplet, airborne routes)	6	1
1.8 Present and recognize the characteristics of a susceptible host	5	1
1.9 Demonstrating an understanding of the importance of screening for infections (e.g. MRSA on admission to hospital, carbapenem resistance for patients with risk factors)	5	2^b^
1.10 Demonstrate the application of standard precautions in healthcare environments	6	1
1.11 Apply appropriate policies/procedures and guidelines when collecting and handling specimens	5	1
1.12 Apply policies, procedures and guidelines relevant to infection control when presented with infection control cases and situations	5	1.25
1.13 Implement work practices that reduce risk of infection (such as taking appropriate immunization or not coming to work when sick to ensure patient and other healthcare worker protection)	6	1
1.14 Appreciate that healthcare workers have the accountability and obligation to follow infection control protocols as part of their contract of employment	6	1
1.15 Act as a role model to healthcare workers and members of the public by adhering to infection prevention and control principles	6	1
1.16 Demonstrating knowledge and awareness of international/national strategies on infection prevention and control and antimicrobial resistance (e.g. Global Action Plan for AMR; WHO SAVE LIVES: Clean Your Hands; UK Government 5-year AMR strategy)	4^a^	2^b^
Domain 2: Antimicrobials and antimicrobial resistance		
2.1 Describe the modes of action of antibiotics and other antimicrobials	4.5^a^	2^b^
2.2 Describe the spectrum of activity for commonly prescribed antimicrobials	5	1
2.3 Describe broad spectrum and narrow spectrum antimicrobials and the contribution of broad-spectrum antimicrobials to antimicrobial resistance	6	1
2.4 Describe the mechanisms of antimicrobial resistance, including: intrinsic or acquired resistance; the importance of selection advantages (e.g. the greater ability for some to colonize) and how this can be an amplification process for antimicrobial resistance	4^a^	1
Domain 3: Antimicrobial prescribing and stewardship		
3.1 Demonstrate an appreciation that appropriate use of antimicrobials reduces the emergence of resistance and reduces adverse effects (e.g. their disruptive effects on host normal flora, which may lead to, for example, *C. difficile* infection, *Candida* spp. infection)	6	1
3.2 Demonstrate an understanding of the key elements of prescribing an antimicrobial, including: obtaining microbiological cultures or other relevant tests before commencing treatment as necessary; the choice of agent; the route of administration; its pharmacokinetics and how this affects the choice of dosage regimen; how to monitor levels and adjust doses (e.g. in the elderly or renal impairment); where to seek specialist advice; decisions to switch agent (e.g. from intravenous to oral, narrower to broader spectrum [or vice versa]) base on microbiological results; the duration of treatment and when to consider review/stop dates	6	0
3.3 Recognize the importance of initiating prompt effective empirical antimicrobial treatment in patients with life-threatening infections (sepsis)	6	0
3.4 Understand why self-limiting bacterial or viral infections are unlikely to benefit from antimicrobials	6	1
3.5 Understand how inflammatory markers and other investigations are used to diagnose and monitor the response to treatment of infections and their complications	5	1
3.6 Describe and demonstrate how to select the appropriate antimicrobial, paying due consideration to local guidance, how, and where, to access this	6	1
3.7 Understand how local microbial/antimicrobial susceptibility patterns impacts on the choice of empirical therapy	5	2^b^
3.8 Demonstrate an understanding of patient specific factors that need to be considered when choosing an antimicrobial which may influence the choice of antimicrobial (i.e. know colonization with resistant organisms)	6	1
3.9 Demonstrate an understanding of how to interpret microbiology results/reports from the laboratory	5	1
3.10 Describe and demonstrate switching to the correct antimicrobial when susceptibility testing indicates resistance, or to a cheaper or more cost-effective antimicrobial that is also compatible with the clinical presentation	6	1
3.11 Describe the common side-effects, including allergy, drug/food interactions, contraindications of the main classes of antimicrobials, and the importance of monitoring for these, and what to do when these are suspected	6	1
3.12 Demonstrate knowledge of when not to prescribe antimicrobials, and use of alternatives, such as the removal of invasive devices (e.g. intravenous or urinary catheters and incision and drainage of abscesses [source control])	6	0.25
3.13 Demonstrate an understanding of the rationale and use of perioperative prophylactic antimicrobials to prevent surgical site infection	5	1
3.14 Demonstrate an understanding of why accurately documenting a patient allergy to an antimicrobial is important	6	0.25
3.15 Demonstrate the importance of documenting in the prescription chart and/or in patients’ clinical records, the clinical indication, route, dose, duration and review date of antimicrobials	6	0.25
3.16 Demonstrate knowledge of when to use a delayed antimicrobial prescription and how to negotiate this with the patient	5	2^b^
3.17 Demonstrate the review of antimicrobial prescriptions for hospital inpatients on all ward rounds. Appropriately choosing one of the five antimicrobial prescribing decisions 48 h after initiating antimicrobial treatment (ARHAI Guidance—Start Smart—then Focus)	6	1
a. Stop antibiotics if there is no evidence of infection		
b. Switch antibiotics from intravenous to oral administration		
c. Change antibiotics—ideally to a narrower spectrum (or broader if required)		
d. Continue and review again at 72 h		
e. Outpatient Parenteral Antibiotic Therapy (OPAT)		
3.18 Educate patients and their carers, nurses and other supporting clinical staff as to when antibiotics are not required and complying with the duration and frequency of administration of their prescribed antimicrobial	5	1.25
Domain 4: Vaccine uptake		
4.1 Able to discuss the relevant national and local immunization programmes and the diseases for which vaccines are currently available. Aware of programmes for specific clinical risk groups and use of vaccination in outbreak situations	5	1
4.2 Able to explain the general principles of immunization (e.g. why multiple and/or booster doses are required, why intervals need to be observed between doses and why the influenza vaccine needs to be given annually)	5	0.25
4.3 Able to clearly and confidently discuss the risks and benefits of vaccination and able to address any concerns patients and/or parents/carers may have	5	1
4.4 Aware of, and able to discuss, any current issues, controversies or misconceptions surrounding immunization	5	1
4.5 Understand how current vaccines can benefit prescribing practices, including reducing the need for prescribing antimicrobials and decreasing antimicrobial-resistant strains (e.g. of *Streptococcus pneumoniae*)	5	2^b^
4.6 Aware of local and national targets for immunization uptake and why vaccine uptake data is important. If appropriate, know where to find data for their area of practice	4^a^	1
Domain 5: Person-centred care		
5.1 Support participation of patients/carers, as integral partners when planning/delivering their care	5.5	1
5.2 Share information with patients/carer in a respectful manner and in such a way that is understandable, encourages discussion, and enhances participation in decision-making	6	1
5.3 Ensure that appropriate education and support is provided by learners to patients/carer, and others involved with their care or service	5	1
5.4 Listen respectfully to the expressed needs of all parties in shaping and delivering care or services	5	2^b^
5.5 Discuss patient/carer expectations or demands of antimicrobials and the need to use antimicrobials appropriately	6	1
Domain 6: Interprofessional collaborative practice		
6.1 Demonstrate an understanding of the roles, responsibilities, and competencies of other health professionals involved in antimicrobial treatment policy decisions	5	2^b^
6.2 Explain why it is important that healthcare professionals, involved in the delivery of antimicrobial therapy (including the prescription, delivery and supply) have a common understanding of antimicrobial treatment policy decisions, the quantity of antimicrobial use, and effective patient/client outcomes	5	1.25
6.3 Establish collaborative communication principles and actively listen to other professionals and patients/carer involved in the delivery of antimicrobial therapy	5	1.25
6.4 Communicate effectively to ensure common understanding of care decisions	5	1
6.5 Develop trusting relationships with patients/carer and other health/social care professionals	5	1.25
6.6 Effectively use information and communication technology to improve interprofessional patient-centred care	5	2^b^

Experts ranked descriptors split into six domains on a six-point Likert scale (1 = strongly disagree; 6 = strongly agree) during the first round of a modified Delphi method questionnaire. Medians and IQRs of responses were calculated.

a Descriptors viewed as less important, i.e. median <5 (on a six-point Likert scale).

b Lack of agreement between experts, i.e. IQR >1.5.

The Joint Information Systems Committee online survey tool was used to develop each round of the online survey, with each survey round open for 3 weeks between May and July 2020. Each expert was emailed a link to complete the first round of the survey. Participants were asked to rank each descriptor on a six-point Likert scale (1 = strongly disagree; 6 = strongly agree) as to the extent to which they assessed it was important to be part of the undergraduate curriculum. An additional open-ended question was included at the end of each domain for experts to include comments, provide feedback and to identify any additional descriptors.

Following the first round the results were analysed by a steering group, ensuring all qualitative feedback was addressed and that no descriptors were unnecessarily excluded. Following analysis of the first-round results, a report was circulated to respondents detailing the quantitative results and inviting further interpretation and feedback. Descriptors for which there was a lack of agreement, descriptors that were amended following feedback and additional descriptors identified by respondents were included in the second-round questionnaire. The second-round questionnaire was sent to all experts who had responded to the first-round survey. Follow-up reminder emails were sent at weekly intervals across the two survey rounds.

### Data analysis

The most frequently used and robust method to determine consensus in Delphi studies is medians and IQRs.^21^ Medians and IQRs were calculated for each descriptor; responses where the median was ≥5 (i.e. experts agreed or strongly agreed with the importance of including) with an IQR ≤1.5 (i.e. there was minimal spread between expert answers) were considered important descriptors that had reached expert consensus.

## Results

### First round

Of the 28 individuals who agreed to participate in the expert panel there was a 100% response rate for completion of the first-round questionnaire. There were high levels of agreement (i.e. median ≥5) on the importance of 51 descriptors (Table 1). Two descriptors were viewed as less important (i.e. median <5) with an IQR of 1, indicating there was a high strength of agreement with minimal spread between expert answers; these two descriptors were excluded: ‘2.4 Describe the mechanisms of antimicrobial resistance, including: intrinsic or acquired resistance and the importance of selection advantages’ and ‘4.6 Aware of local and national targets for immunization uptake and why vaccine uptake data is important. If appropriate, know where to find data for their area of practice’. Additionally, two descriptors were viewed as less important but with a low strength of agreement between experts: ‘1.16 Demonstrating knowledge and awareness of international/national strategies on infection prevention and control and antimicrobial resistance (e.g. Global Action Plan for AMR; WHO SAVE LIVES: Clean Your Hands; UK Government 5-year AMR strategy)’ and ‘2.1 Describe the modes of action of antibiotics and other antimicrobials’. These 2 descriptors, and a total of 10 descriptors with a low strength of agreement between experts (i.e. IQR >1.5) were included in the second-round survey. Of the initial 55 descriptors, 43 reached consensus (i.e. median ≥5 and IQR ≤1.5) by the expert panel (78%). The 12 descriptors which had disagreement, 4 amended descriptors and 12 new descriptors identified following qualitative feedback formed the second round of the survey (Table 2).

**Table 2.** Round two responses

New descriptors	Median	IQR
Domain 1: Infection prevention and control		
Aware of which vaccinations healthcare workers should receive in addition to standard UK immunizations	5	1
Describe what is meant by contact precautions, droplet precautions and airborne precautions	6	1
Awareness of the cost (e.g. to the patient, society, healthcare system) of hospital acquired infections	5	2^b^
Understand how to use PPE and when to apply to appropriate situations	6	1
Domain 2: Antimicrobials and antimicrobial resistance		
Describe the implications of commonly encountered resistance profiles in terms of patient management (e.g. MRSA, VRE, ESBL, CPE)	5	1
Awareness of factors contributing to AMR including inappropriate prescribing by healthcare workers and the sale of antimicrobials without prescription (e.g. over the counter in some parts of the world; online sales)	5	1
Understand the link between antimicrobials and the human microbiome and how this facilitates spread of resistant organisms	5	2^b^
Aware of which vaccinations healthcare workers should receive in addition to standard UK immunizations	4^a^	1
Domain 3: Antimicrobial prescribing and stewardship		
Describe key features of specific infections and the best narrow spectrum antibiotics to prescribe and length of antibiotic course in these scenarios (e.g. UTI, pneumonia, cellulitis)	6	1
Understand how to request and interpret basic diagnostic tests that can guide antimicrobial therapy (e.g. microbiology, radiology, immunology)	6	0.25
Domain 4: Vaccine uptake		
Knowledge of different types of vaccine and different vaccine development strategies	4^a^	1
Understand cultural sensitivities around refusal to take vaccines	5	1

Round one descriptors	Median	IQR

Domain 1: Infection prevention and control		
1.4 Explain the ‘Chain of Infection’	5	1
1.16 Demonstrating knowledge and awareness of international/national strategies on infection prevention and control and antimicrobial resistance (e.g. Global Action Plan for AMR; WHO SAVE LIVES: Clean Your Hands; UK Government 5-year AMR strategy)	4^a^	1
1.9R Demonstrate an understanding of the principles of why screening for infections (e.g. MRSA on admission to hospital) is important for reducing nosocomial spread	5.5	1
Domain 2: Antimicrobials and antimicrobial resistance		
2.1R Demonstrate an understanding of the spectrum of antibiotic activity in terms of Gram-positive, Gram-negative, anaerobic and atypical organisms.	6	1
Domain 3: Antimicrobial prescribing and stewardship		
3.7 Understand how local microbial/antimicrobial susceptibility patterns impacts on the choice of empirical therapy	5	1
3.16 Demonstrate knowledge of when to use a delayed antimicrobial prescription and how to negotiate this with the patient	5	1
3.18R Demonstrate the ability to educate patients and their carers, nurses and other supporting clinical staff about when antibiotics are and are not required, the importance of complying with the duration/frequency of administration of their prescribed antimicrobial and when to seek help	6	1
Domain 4: Vaccine uptake		
4.5 Understand how current vaccines can benefit prescribing practices, including reducing the need for prescribing antimicrobials and decreasing antimicrobial-resistant strains (e.g. of *S. pneumoniae*)	5	2^b^
Domain 5: Person-centred care		
5.4 Listen respectfully to the expressed needs of all parties in shaping and delivering care or services	5.5	1
Domain 6: Interprofessional collaborative practice		
6.1 Demonstrate an understanding of the roles, responsibilities, and competencies of other health professionals involved in antimicrobial treatment policy decisions	5	1
6.6 Effectively use information and communication technology to improve interprofessional patient-centred care	5	2^b^
6.2R Explain why it is important that healthcare professionals involved in the delivery of antimicrobial therapy (including the prescription, delivery and supply) have a common understanding of antimicrobial treatment policy decisions, the quantity/quality of antimicrobial use, and effective patient/client outcomes	5	1.25

Experts ranked descriptors split into six domains on a six-point Likert scale (1 = strongly disagree; 6 = strongly agree) during the second round of a modified Delphi method questionnaire. The second-round questionnaire included 12 new descriptors, 4 amended round-one descriptors (R) and 12 round-one descriptors with disagreement. Medians and IQRs of responses were calculated.

PPE, personal protective equipment.

a Descriptors viewed as less important, i.e. median <5 (on a six-point Likert scale).

b Lack of agreement between experts, i.e. IQR >1.5.

### Second round

All 28 members of the expert panel were invited to complete the second-round questionnaire; there was a 100% response rate to the second round. Three descriptors were viewed as less important, with minimal spread between expert answers (i.e. median <5 and IQR <1.5) and were excluded (Table 2). These included one descriptor that had not reached consensus in the first round and two new descriptors: ‘1.16 Demonstrating knowledge and awareness of international/national strategies on infection prevention and control and antimicrobial resistance (e.g. Global Action Plan for AMR; WHO SAVE LIVES: Clean Your Hands; UK Government 5-year AMR strategy)’, ‘Knowledge of how different global settings have inadvertently led to the development of resistance (e.g. sewage contamination of water sources in India led to NDM)’ and ‘Knowledge of different types of vaccine and different vaccine development strategies’. The strength of agreement between experts was high for 19 descriptors and low for 4 descriptors. Of the 24 descriptors that formed the second round, 17 reached consensus (71%).

### Summary statement

We have identified 58 competency descriptors within six domains that have reached expert consensus by a group representing UK medical schools from England, Wales and Northern Ireland. These competencies form an AMR and AMS framework for undergraduate medical student education (Appendix S2).

## Discussion

Utilizing expert opinion from across the UK, we present here the first set of specific AMR/S competencies for UK undergraduate medical student education. This framework can be used to inform standards for education and prescribing and will help standardize a high-level of antimicrobial knowledge for tomorrow’s doctors. The competencies defined here have been developed to address a gap in UK undergraduate medical student education and to ensure that all new graduates are trained in the principles of evidence based AMS. In the EU alone, it is estimated that infections from MDR bacteria result in 25 000 deaths annually.^3^ Hence, it is not surprising that medical students perceive misuse of antibiotics as unethical.^13^ Castro-Sánchez *et al*.^22^ identified that UK medical school courses contain a median of 17.8 h (IQR 8.87–42.62) of AMS education, with only 16 of 23 medical schools (69.5%) teaching all recommended AMS principles when assessing 2012 curricula.

Evidence shows that many medical students lack self confidence in choosing the correct antibiotics, deciding when to use combination therapy and choosing the correct dose and interval of administration, with up to 98% of students wanting more training on antibiotic use during medical school.^12–18^^,^^23–27^ A study of self-reported preparedness for prudent antibiotic use among final-year medical students in 29 European countries reported that UK students felt least prepared on selecting initial empirical therapy without using guidelines.^27^ This further highlights gaps in UK undergraduate medical education, with an overreliance on guidelines rather than a fundamental understanding of how to select appropriate antibiotics for common infections. A survey of junior doctors in the UK and France identified gaps in knowledge on the prevalence of antibiotic resistance and antibiotic misuse, despite 98.6% of those surveyed having prescribed an antibiotic within the last 6 months.^28^

To improve AMS in the UK a coherent and consistent national approach must be taken to create high-quality educational resources to train current and future healthcare workers and improve individual practice. Using predefined competency statements with associated descriptors, experts representing medical schools in the UK formed a consensus on core AMR/S competencies for UK undergraduate medical students. There was a high response rate to both rounds of the Delphi process, with consistently high levels of agreement for many descriptors. Within the overarching domains of: ‘Infection prevention and control’, ‘Antimicrobials and antimicrobial resistance’, ‘Antimicrobial prescribing and stewardship’, ’Vaccine uptake’, ‘Person-centred care’ and ‘Interprofessional collaborative practice’ we have reached consensus on 58 competency descriptors. These competencies can be used by professional bodies, regulators and education providers to inform standards, design curricula, create teaching materials and assess learning outcomes.

Strengths of the study include the use of a robust methodology, a high response rate and the opinions of a defined panel of experts. Some may consider the professional background of the experts in this study, including mostly specialists in infectious diseases or microbiology, a limitation in reaching consensus on relevance to all prescribers. However, experts in this group were also selected due to their role in undergraduate infection teaching and are therefore likely to have a pragmatic understanding of expectations and limitations of what can be included within undergraduate medical student education. In addition, the competency frameworks used to form the first-round survey had involved much broader input from other healthcare professionals (e.g. dentists, nurses, midwives, pharmacists), allowing the specialist expert group here to prioritize specific competencies for future doctors.

Preventing the rise of AMR requires multifactorial interventions and collaboration between healthcare professionals. It is essential and urgent that AMR and AMS competencies are embedded into the curricula of all healthcare professionals, including medical students, and we encourage those that develop curricula to adopt a similar process to develop competencies specific to their professional group.

## Supplementary Material

dlaa096_Supplementary_Data
