# Supplementary material for: Consensus-based antimicrobial resistance and stewardship competencies for UK undergraduate medical students
Source: JAC Antimicrob Resist. 2020 Dec 4;2(4):dlaa096. doi: 10.1093/jacamr/dlaa096 (PMC8210211; doi:10.1093/jacamr/dlaa096)
Supplement: dlaa096_Supplementary_Data [file dlaa096_supplementary_data.docx]

**Supplementary data**

**Appendix S1.** UK medical schools involved in the development of a consensus-based antimicrobial resistance and stewardship framework for undergraduate medical education.

| Aston University Medical School |
| --- |
| Barts and the London School of Medicine and Dentistry |
| Brighton and Sussex Medical School |
| Cardiff University School of Medicine |
| Hull York Medical School |
| Imperial College London Faculty of Medicine |
| King’s College London GKT School of Medical Education |
| Newcastle University School of Medical Education |
| Queen’s University Belfast School of Medicine |
| St George’s, University of London |
| Swansea University Medical School |
| University College London Medical School |
| University of Birmingham College of Medical and Dental Sciences |
| University of Bristol Medical School |
| University of Cambridge School of Clinical Medicine |
| University of Leeds School of Medicine |
| University of Leicester Medical School |
| University of Liverpool School of Medicine |
| University of Manchester Medical School |
| University of Nottingham School of Medicine |
| University of Oxford Medical Sciences Division |
| University of Sheffield Medical School |
| University of Southampton School of Medicine |
| University of Sunderland School of Medicine |
| University of Warwick Medical School |

**Appendix S2.** Antimicrobial resistance and stewardship framework for UK undergraduate medical education.

| **Domain 1: Infection prevention and control** |
| --- |
| *Competency statement*  All newly qualified doctors must understand the core knowledge underpinning infection prevention and control and use this knowledge appropriately to prevent the spread of infection. |
| *Descriptors*  1. Describe the nature and classification of pathogenic micro-organisms  2. Describe how micro-organisms cause infections in humans: the importance of understanding the differences between colonisation (e.g. of venous leg ulceration) and infection  3. Explain what an antimicrobial resistant organism is  4. Explain the ‘Chain of Infection’  5. Describe how micro-organisms are transmitted in both community and hospital settings  6. Define the components required for infection transmission (i.e. presence of an organism, route of transmission of the organism from one person to another, a host who is susceptible to infection)  7. Describe the routes of transmission of infectious organisms (i.e. contact, droplet, airborne routes)  8. Present and recognise the characteristics of a susceptible host.  9. Demonstrate an understanding of the principles of why screening for infections (e.g. MRSA on admission to hospital) is important for reducing nosocomial spread  10. Demonstrate the application of standard precautions in healthcare environments  11. Apply appropriate policies/procedures and guidelines when collecting and handling specimens  12. Apply policies, procedures and guidelines relevant to infection control when presented with infection control cases and situations  13. Implement work practices that reduce risk of infection (such as taking appropriate immunisation or not coming to work when sick to ensure patient and other healthcare worker protection)  14. Appreciate that healthcare workers have the accountability and obligation to follow infection control protocols as part of their contract of employment  15. Act as a role model to healthcare workers and members of the public by adhering to infection prevention and control principles  16. Demonstrate awareness of which vaccinations healthcare workers should receive in addition to standard UK immunisations  17. Describe what is meant by contact precautions, droplet precautions and airborne precautions  18. Understand how to use PPE and when to apply to appropriate situation |
| **Domain 2: Antimicrobials and antimicrobial resistance** |
| *Competency statement*  All newly qualified doctors need to understand the core knowledge underpinning the action of antibiotics and use this knowledge to help prevent antimicrobial resistance. |
| *Descriptors*  1. Demonstrate an understanding of the spectrum of antibiotic activity in terms of Gram positive, Gram negative, anaerobic and atypical organism  2. Describe the spectrum of activity for commonly prescribed antimicrobials  3. Describe broad spectrum and narrow spectrum antimicrobials and the contribution of broad-spectrum antimicrobials to antimicrobial resistance  4. Describe the implications of commonly encountered resistance profiles in terms of patient management (e.g. MRSA, VRE, ESBL, CPE)  5. Awareness of factors contributing to AMR including inappropriate prescribing by healthcare workers and the sale of antimicrobials without prescription (e.g. over the counter in some parts of the world; online sales) |
| **Domain 3: Antimicrobial prescribing and stewardship** |
| *Competency statement*  All newly qualified doctors need to demonstrate knowledge in how infections are diagnosed and managed and use this knowledge appropriately to manage patients with infections including the appropriate use of antimicrobial agents. |
| *Descriptors*  1. Demonstrate an appreciation that appropriate use of antimicrobials reduces the emergence of resistance and reduces adverse effects (e.g. their disruptive effects on host normal flora, which may lead to, for example, C. difficile infection, Candida spp infection)  2. Demonstrate an understanding of the key elements of prescribing an antimicrobial, including: obtaining microbiological cultures or other relevant tests before commencing treatment as necessary; the choice of agent; the route of administration; its pharmacokinetics and how this affects the choice of dosage regimen; how to monitor levels and adjust doses (e.g. in the elderly or renal impairment); where to seek specialist advice; decisions to switch agent (e.g. from intravenous to oral, narrower to broader spectrum [or vice versa]) base on microbiological results; the duration of treatment and when to consider review/stop dates  3. Recognise the importance of initiating prompt effective empirical antimicrobial treatment in patients with life-threatening infections (sepsis)  4. Understand why self-limiting bacterial or viral infections are unlikely to benefit from antimicrobials  5. Describe key features of specific infections and the best narrow spectrum antibiotics to prescribe and length of antibiotic course in these scenarios (e.g. UTI, pneumonia, cellulitis)  6. Understand how inflammatory markers and other investigations are used to diagnose and monitor the response to treatment of infections and their complications  7. Understand how to request and interpret basic diagnostic tests that can guide antimicrobial therapy (e.g. microbiology, radiology, immunology)  8. Describe and demonstrate how to select the appropriate antimicrobial, paying due consideration to local and national guidance, how, and where, to access this  9. Understand how local microbial/antimicrobial susceptibility patterns impacts on the choice of empirical therapy  10. Demonstrate an understanding of patient specific factors that need to be considered when choosing an antimicrobial which may influence the choice of antimicrobial (i.e. know colonisation with resistant organisms)  11. Demonstrate an understanding of how to interpret microbiology results/reports from the laboratory  12. Describe and demonstrate switching to the correct antimicrobial when susceptibility testing indicates resistance, or to a cheaper or more cost-effective antimicrobial that is also compatible with the clinical presentation  13. Describe the common side-effects, including allergy, drug/food interactions, contraindications of the main classes of antimicrobials, and the importance of monitoring for these, and what to do when these are suspected  14. Demonstrate knowledge of when not to prescribe antimicrobials, and use of alternatives, such as the removal of invasive devices (e.g. intravenous or urinary catheters and incision and drainage of abscesses [source control]).  15. Demonstrate an understanding of the rationale and use of perioperative prophylactic antimicrobials to prevent surgical site infection  16. Demonstrate an understanding of why accurately documenting a patient allergy to an antimicrobial is important  17. Demonstrate the importance of documenting in the prescription chart and/or in patients’ clinical records, the clinical indication, route, dose, duration and review date of antimicrobials  18. Demonstrate knowledge of when to use a delayed antimicrobial prescription and how to negotiate this with the patient  19. Demonstrate the review of antimicrobial prescriptions for hospital inpatients on all ward rounds. Appropriately choosing one of the five antimicrobial prescribing decisions 48 hours after initiating antimicrobial treatment (ARHAI Guidance – Start Smart – then Focus)  a. Stop antibiotics if there is no evidence of infection  b. Switch antibiotics from intravenous to oral administration  c. Change antibiotics – ideally to a narrower spectrum (or broader if required)  d. Continue and review again at 72 hours  e. Outpatient Parenteral Antibiotic Therapy (OPAT)  20. Demonstrate the ability to educate patients and their carers, nurses and other supporting clinical staff about when antibiotics are and are not required, the importance of complying with the duration/frequency of administration of their prescribed antimicrobial and when to seek help |
| **Domain 4: Vaccine uptake** |
| *Competency statement*  All newly qualified doctors need to demonstrate knowledge in the importance of vaccines in reducing antimicrobial resistance and use this knowledge appropriately to promote vaccination. |
| *Descriptors*  1. Demonstrate the ability to discuss the relevant national and local immunisation programmes and the diseases for which vaccines are currently available. Aware of programmes for specific clinical risk groups and use of vaccination in outbreak situations  2. Demonstrate the ability to explain the general principles of immunisation (e.g. why multiple and/or booster doses are required, why intervals need to be observed between doses and why the influenza vaccine needs to be given annually)  3. Demonstrate the ability to clearly and confidently discuss the risks and benefits of vaccination and able to address any concerns patients and/or parents/carers may have  4. Demonstrate awareness of, and ability to discuss, any current issues, controversies or misconceptions surrounding immunisation  5. Demonstrate awareness of the cultural sensitivities around refusal to take vaccines |
| **Domain 5: Person centred care** |
| *Competency statement*  All newly qualified doctors must seek out, integrate and value as a partner the input and engagement of the patient /carer in designing and implementing care. |
| *Descriptors*  1. Support participation of patients/carers as integral partners when planning/delivering their care  2. Share information with patients/carers in a respectful manner and in such a way that is understandable, encourages discussion, and enhances participation in decision-making. This includes discussing with the patient/carers about what to do if their condition deteriorates (safety-netting advice)  3. Ensure that appropriate education and support is provided by learners to patients/carers, and others involved with their care or service  4. Listen respectfully to the expressed needs of all parties in shaping and delivering care or services  5. Discuss patient/carers expectations or demands of antimicrobials and the need to use antimicrobials appropriately |
| **Domain 6: Interprofessional collaborative practice** |
| *Competency statement*  All newly qualified doctors need to understand how different professions collaborate in relation to how they contribute to AS. |
| *Descriptors*  1. Demonstrate an understanding of the roles, responsibilities, and competencies of other health professionals involved in antimicrobial treatment policy decisions  2. Explain why it is important that healthcare professionals involved in the delivery of antimicrobial therapy (including the prescription, delivery and supply) have a common understanding of antimicrobial treatment policy decisions, the quantity/quality of antimicrobial use, and effective patient/client outcomes  3. Establish collaborative communication principles and actively listen to other professionals and patients/carers involved in the delivery of antimicrobial therapy  4. Communicate effectively to ensure common understanding of care decisions  5. Develop trusting relationships with patients/carer and other health/social care professionals |
